
JOURNAL INFORMATION
==============================
Journal ID: ijcoms

IJQHC Communications

EISSN: 2634-5293

ARTICLE INFORMATION
==============================
PMCID: PMC9384607
DOI: 10.1093/ijcoms/lyac011
Article ID: lyac011
Article version: 1
Subjects: Perspective, AcademicSubjects/MED00862

Trust as a Factor for Higher Performance in Health Care: COVID 19, Digitalization, and Positive Patient Experiences

Cado Vesna International University of Monaco, Eye Hospital Sistina Ophthalmology

Corresponding Author: Vesna Cado, email: vesna.cado@gmail.com
Electronic publication date: 2022 Jul 30
Electronic Location ID: lyac011
Received 2021 Oct 17; Revised 2022 Jun 28; Accepted 2022 Jul 29; 2022 Jul 26
Copyright: © The Author(s) 2022. Published by Oxford University Press on behalf of International Society for Quality in Health Care.
Copyright year: 2022
License: This is an Open Access article distributed under the terms of the Creative Commons Attribution License (https://creativecommons.org/licenses/by/4.0/), which permits unrestricted reuse, distribution, and reproduction in any medium, provided the original work is properly cited.
License URL: https://creativecommons.org/licenses/by/4.0/

edited-state: accepted-manuscript
article-lifecycle: PAP

==============================

### Trust as a factor for higher performance in health care: COVID 19, digitalization, and positive patient experiences by

Vesna Cado, MPPM, DBA
International University of Monaco
Eye Hospital Sistina Ophthalmology
email: vcado@monaco.edu; vesna.cado@gmail.com

ACCEPTED MANUSCRIPT

### Trust as a factor for higher performance in healthcare:

### COVID-19, digitalization, and positive patient experiences

**When people trust, they commit.**
**When committed, they are efficient.**
**When efficient, goals are attained.**

Hospitals that build a culture of trust with their employees, patients, and wider communities create long-term relationships and improve the competitive edge of their business. One of the most used definitions of trust defines the concept as confidence in the reliability and integrity of the exchange partner [1]. In their definition, Morgan and Hunt [1], associated trust with helpfulness, competency, responsibility, benevolence, honesty, and fairness as its vital components. Other researchers further define trust as a company's ability to respond to consumer needs in risky situations [2]. Companies that will put the interest and welfare of their consumers, employees, and wider community before their profits, particularly in times of healthcare crises, earn their trust and loyalty.

A recent study published in the Harvard Business Review (HBR) [3] found that high-trust organizations have stronger performance due to higher employee productivity, a climate of

© The Author(s) 2022. Published by Oxford University Press on behalf of International Society for Quality in Health Care.

positive energy, and willingness to collaborate to attain common organizational goals. Study reports that compared people at low-trust companies with people at high-trust companies showed that the latter had 74% less stress, 106% more energy at work, 50% higher productivity, 13% fewer sick days, 76% more engagement, 29% more satisfaction with their lives, and 40% less burnout. A global survey conducted by PricewaterhouseCoopers (PwC) in 2016 reported that 55% of chief executive officers (CEOs) have found that a lack of trust is a threat to their organisation's growth [4].

In healthcare, creating a safe, supportive, and compassionate atmosphere is essential for making a personal connection with patients, fostering trust and collaboration, and delivering better quality medical care [5]. Trust between the patient and the medical provider (doctors, nurses, administrators) influences patient management outcomes, particularly in regard to the treatment of long-term illness and prevention initiatives. Dr J.R. Baron, CEO of the American Board of Internal Medicine (ABIM) [6] says,

> *It was patient's first visit to my office, and I was 30 minutes late. By the time I saw her, she was understandably furious. Instead of being defensive, I apologized sincerely. She calmed down. We were able to start over and tackle the issue that brought her in. For 20 years, we worked together in sickness and health, as patient and physician. That encounter taught me that people need to be respected and heard in the doctor's office or the trust needed to care for them will never develop.*

Trust requires healthcare providers to devote themselves to and act toward patients in the same way that they would like to be treated. Patients are 'vulnerable customers' who want to be heard, understood, and engaged. Hospitals that develop strategies to meet the emotional and physical needs of their patients by practising the patient experience philosophy gain patients' trust and higher business performance. As the famous American poet Maya Angelou

ACCEPTED MANUSCRIPT

says, ‘People will forget what you said and did, but they will never forget how you made them feel’ [7].

Therefore, we discuss several important aspects of trust.

**Trust during COVID-19**

Trust is an essential factor, especially during the COVID-19 pandemic. Patients closely watch and assess how hospitals cope with crises. They decide to grant their trust based on competence (delivering on promises), ethical behaviour, and social responsibility. Therefore, hospitals need to focus on the timely and transparent representation of security measures, safety protocols, and a caring culture to retain patient loyalty and ensure the hospital’s sustainability. A recent case study [8] conducted on best practices at an eye hospital in Skopje, Macedonia, Sistina Ophthalmology during COVID-19, showed that with an emergency plan focused on human experiences that was promptly executed at the beginning of the crisis, the eye hospital succeeded in keeping safe and satisfying its employees and retaining 40% of patients. A 57-year-old hospital patient, Ana, shares her experience on trust:

> *I knew that I could trust you; you are a serious hospital, especially important in this troubled situation. The extra effort you have made - but did not have to - meant the world to me. On the day of my operation, I was offered to use your free transport patient service, as explained, for those in need during the crises. It was so clean, and with all disinfection measures undertaken, I felt safe. I really felt special! I did not expect such a gesture. Although on the day of the*

ACCEPTED MANUSCRIPT

*surgery I felt really scared, such a devoted and caring approach has helped me a lot to overcome my anxiety.*

According to the 2020 Edelman Trust Barometer [9], during COVID-19, organizations may build a new level of connection with customers or lose the relationship forever. Over one-third of customers prioritized new brands only because of their innovative and compassionate responses to the crisis. In contrast, the report states that 71% of customers say that if they perceive that a brand puts profit over people, they will lose trust in that brand forever.

This is also reflected in employees' need for physical and emotional protection in the workplace and society. The emotional, mental, and financial support that hospitals offer to their medical and administrative staff as well as the wider community build trusting relationships and ensure employee loyalty and dedication to their work. Mindfulness training, yoga sessions, and monthly meetings such as Schwartz Rounds allow employees to share their emotions, reduce their stress levels, and positively support their well-being [10]. According to a recent study on the Google Corporation [11], a positive work environment in which managers express concern for employees' personal well-being stimulates higher organizational performance.

**Can trust be digitalized in healthcare?**

Digitalization represents both a threat and a future opportunity, particularly for human interactions. Hospitals are forced to reinvent their operational and communication strategies to simultaneously address the physical and emotional needs of patients, employees, and the wider community at the same time. Digital care and physician virtual visits received a tremendous boost at the beginning of the COVID-19 crisis. The pandemic seized interactions and patient journey through a focal point of 10–15 minutes, hampered the insufficient digital

ACCEPTED MANUSCRIPT

skills of caregivers, questioned the types of digital health services, challenged patients' trust regarding the use of digital platforms, and disrupted the availability of digital devices for vulnerable groups of patients [12]. In spite of the difficulties in its implementation, digitalization has offered many advantages. Lessons learned from other already-digitalized industries, such as financial, retail, and travel services, may be beneficial to healthcare providers and lead to positive patient experiences. Healthcare providers are now offering numerous mobile device-based digital services to schedule appointments, locate doctors, renew prescriptions, and monitor patients' conditions. Virtual care models have thus far primarily been applicable in primary care settings. However, continuous investment in digitalization, which is required to sustain the changes that came about during the COVID-19 pandemic, creates new digital tools to help patients in acute-care settings. A large survey study [13] published in 2021 in the Journal of Medical Internet Research that assessed patients' experiences with virtual visits in a diverse patient population found that patient-clinician engagement in virtual visits is comparable to such engagement during in-person visits and has a high patient experience score. Additionally, research on the quality of virtual care conducted in 2021 by Virginia Commonwealth University Health [14] confirmed the positive effects of digitalization on performance in healthcare by showing that virtual care through telepsychiatry reduced missing appointments by 5% compared to before the COVID-19 pandemic. Healthcare providers must consider virtual care a serious option for building trustful relationships with their patients and improving their efficiency.

ACCEPTED MANUSCRIPT

**Future of trust in healthcare**

If trends in technological advancements continue as expected, digital empathy and trusting relationships in healthcare will become an even more critical issue. Hospitals will need to allocate more resources to user-friendly technologies, tools, and the skills of their personnel to better facilitate digital communication with patients, showing understanding and emotional attachment in delivering professional medical support and continuity of care.

Higher performance in healthcare will also depend on providers' management capacity to build a highly trustworthy organization as well as the ability to cultivate trust within their system. To do so, they will need to encourage, engage, and empower their employees by providing clear directions and giving them the resources and freedom to decide how to execute a project in their own way while also holding them accountable for their decisions.

Rapid and advanced use of innovative digital tools seriously endangers facets of trusting relationships through the mechanisms of privacy, security, and accountability, which are particularly important aspects of healthcare. HBR [15] reports on community-based joint efforts to build a new global architecture for security that is capable of responding to today's digital reality. Governments, corporations, and other actors should invest energy and resources to fight large-scale hacking and the tracking of digital activities and locations as well as to control the security of the valuable resources and data available to malicious actors. Authorities will also need to create legal frameworks that hold businesses and providers accountable for their identity and operations and protect employees and patients. The new reality will bring about new frontiers for quality and competitiveness for all actors involved in healthcare.

ACCEPTED MANUSCRIPT

**References**:

[1] Morgan RM, Hunt SD. The commitment-trust theory of relationship marketing. Journal of Marketing. 1994 Jul;58(3):20-38.

[2] Delgado-Ballester E, Munuera-Aleman JL, Yague-Guillen MJ. Development and validation of a brand trust scale. International Journal of Market Research. 2003 Jan 1;45(1):35-54.

[3] The neuroscience of trust [Internet]. Harvard Business Review. HBR. 2021 [cited 2021 Nov 24]. Available from: https://hbr.org/2017/01/the-neuroscience-of-trust

[4] PWC 19th annual global CEO survey - PWC: Audit and ... [Internet]. PWC [cited 2021 Nov 24]. Available from: https://www.pwc.com/gx/en/ceo-survey/2016/landing-page/pwc-19th-annual-global-ceo-survey.pdf

[5] Lee TH. How US health care got safer by focusing on the patient experience. Harvard Business Review. 2017 May.

[6] Baron RJ. Building trust can improve American healthcare [Internet]. AJMC [cited 2021 Nov 24]. Available from: https://www.ajmc.com/view/building-trust-can-improve-american-health care

[7] Neubauer CE, Angelou M. An interview with Maya Angelou. The Massachusetts Review. 1987 Jul 1;28(2):286-92.

[8] Cado V. Positive patient experience in eye care during COVID-19: A case from Eye Hospital Sistina Oftalmologija. Patient Experience Journal. 2020;7(2):103-11.

[9] Barometar ET. Maintaining brand trust during the coronavirus - Edelman [Internet]. [cited 2021 Nov 24]. Available from: https://www.edelman.com/sites/g/files/aatuss191/files/2020-03/2020%20Edelman%20Trust%20Barometer%20Brands%20and%20the%20Coronavirus.pdf

[10] Lown BA, Manning CF. The Schwartz Center rounds: Evaluation of an interdisciplinary approach to enhancing patient-centered communication, teamwork, and provider support. Academic Medicine. 2010 Jun 1;85(6):1073-81.

ACCEPTED MANUSCRIPT

[11] Pfeffer J, Sutton RI. How Google sold its engineers on management [Internet]. Harvard Business Review. 2019 [cited 2021 Nov 24]. Available from: https://hbr.org/2013/12/how-google-sold-its-engineers-on-management

[12] Kaihlanen AM, Virtanen L, Buchert U, Safarov N, Valkonen P, Hietapakka L, Hörhammer I, Kujala S, Kouvonen A, Heponiemi T. Towards digital health equity- A qualitative study of the challenges experienced by vulnerable groups in using digital health services in the COVID-19 era. BMC Health Services Research. 2022 Dec;22(1):1-2.

[13] Rose S, Hurwitz HM, Mercer MB, Hizlan S, Gali K, Yu PC, Franke C, Martinez K, Stanton M, Faiman M, Rasmussen P. Patient experience in virtual visits hinges on technology and the patient-clinician relationship: A large survey study with open-ended questions. Journal of Medical Internet Research. 2021 Jun 21;23(6):e18488.

[14] Virginia Commonwealth University Health. Telepsychiatry ... [Internet]. AMA. [cited 2021 Nov 24]. Available from: https://www.ama-assn.org/system/files/2021-05/case-study-virginia-commonwealth-university-health-telepsychiatry-during-covid-19.pdf

[15] Why companies are forming cybersecurity alliances [Internet]. Harvard Business Review. 2021 [cited 2021 Nov 24]. Available from: https://hbr.org/2019/09/why-companies-are-forming-cybersecurity-alliances

ACCEPTED MANUSCRIPT
